# Management of a Symphyseal Multilocular Odontogenic Keratocyst

**DOI:** 10.14740/jmc5286

**Published:** 2026-06-03

**Authors:** Laurianne Giraudeau, Bruno Meymandi-Nejad, Philippe Alessi, Stephanie Carier, Anne Vidil, Fredric Denis, Laurent Estrade

**Affiliations:** a Oral Surgery, Private Practice, Chateauroux 36000, France; b Dental Surgery, Private Practice, Chateauroux 36000, France; c Orthodontist, Private Practice, Mereau 18120, France; d Maxilofacial Surgery, Private Practice, Bordeaux 33000, France; e Orthopedic Surgery, Private Practice, La Seyne-sur-Mer 83500, France; f Department of Oral Medicine and Surgery, Tours University Hospital, Tours 37000, France

**Keywords:** Odontogenic keratocyst, Multilocular, Symphyseal, Sticky bone, Platelet-rich fibrin, *Actinomyces odontolyticus*

## Abstract

Multilocular odontogenic keratocyst (MOK) is a benign odontogenic lesion with significant aggressive behavior and a high risk of recurrence. Its occurrence in the symphyseal region is rare, with the posterior mandible being the usual site of predilection. We report the case of an MOK diagnosed incidentally in a 30-year-old female patient. Treatment consisted of endodontic therapy, complete excision via a vestibular approach, and immediate reconstruction using an autologous composite biomaterial known as “sticky bone,” supplemented with platelet-rich fibrin membranes. Bacteriological analysis revealed a secondary infection with *Actinomyces odontolyticus*, requiring a 3-month course of prolonged antibiotic therapy. At 3 months, follow-up examinations showed homogeneous ossification, preservation of the inferior alveolar nerve, and no evidence of recurrence. Given the high risk of recurrence, conservative marginal resection combined with the use of sticky bone allowed for reliable reconstruction. The addition of platelet-rich fibrin contributed to enhanced healing and bone maturation. The anatomical and functional outcomes are satisfactory, although long-term follow-up remains necessary. This case highlights several important clinical and therapeutic points. MOKs may be discovered incidentally and require careful radiological and histopathological evaluation for accurate diagnosis and appropriate management. Conservative marginal resection, when combined with modern biomaterials such as sticky bone, can achieve effective bone reconstruction while minimizing surgical morbidity. In addition, the adjunctive use of platelet-rich fibrin enhances soft tissue healing and promotes bone regeneration. Clinicians should also be aware that secondary infection, such as *Actinomyces odontolyticus*, may occur and can require prolonged, targeted antibiotic therapy. Finally, close multidisciplinary coordination among practitioners is essential to optimize sequential management and to reduce the need for more invasive surgical procedures.

## Introduction

Odontogenic keratocyst (OK), formerly known as the “keratocystic odontogenic tumor,” is a benign odontogenic lesion characterized by a parakeratinized epithelial lining. This type of lesion shows a tendency for intraosseous expansion as well as a high recurrence rate [1]. Another form of OK, known as orthokeratinized odontogenic cyst, associated with an impacted tooth in 50% of case, could be confounded with a dentigerous cyst, as it presents as a unilocular radiographic lesion, and is less likely to recur.

We present a case of a radiologically multiloculated form of OK, the multilocular odontogenic keratocyst (MOK), occurring in the symphyseal region. MOK is an odontogenic lesion with potentially aggressive behavior and a high risk of recurrence, reported between 12% and 58% depending on the series, particularly in the presence of satellite microcysts, thin cyst walls, or cortical perforations [2]. According to the 2022 World Health Organization (WHO) classification, multilocular forms do not represent a specific histopathological subtype but rather correspond solely to a radiological appearance, which may also include unilocular lesions and the term “primordial cyst” is no longer considered synonymous with OK and is now recognized as a distinct pathological entity [3]. The type of OK in the symphyseal region is rare, as OKs most commonly arise in the posterior mandible [4, 5]. The presented case illustrates the diagnostic and therapeutic challenges posed by an anterior MOK combined with an unusual chronic intracystic superinfection due to *Actinomyces odontolyticus*. The therapeutic challenge involved, on the one hand, the bone resection of this lesion (marginal or *en bloc*), and on the other hand, the reconstruction of the significant bone defect.

## Case Report

A 30-year-old woman, otherwise healthy, was referred for evaluation of a multilocular radiolucent mandibular lesion discovered incidentally during an orthodontic assessment. Clinically, there was moderate flattening of the labiomental fold without lymphadenopathy or cutaneous inflammation. The floor of the mouth was soft and non-indurated, though slight vestibular swelling was noted. The patient also reported intermittent episodes of bilateral chin paresthesia.

**Figure 1:**
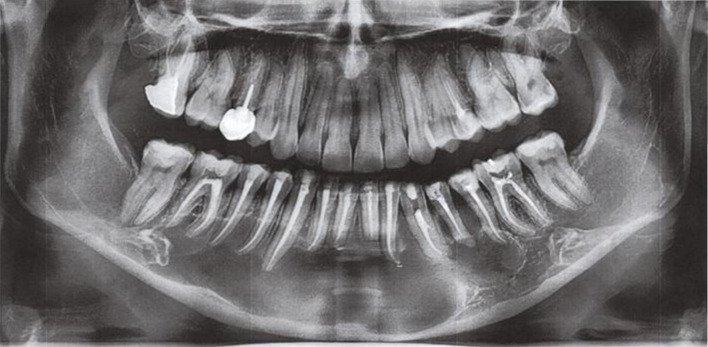
Orthopantomogram.

Cone beam computed tomography (CBCT) confirmed the multilocular nature of this expansive lesion centered on the mandibular symphysis, extending from teeth 35 to 46, consistent with the orthopantomogram findings (Fig. 1). The lesion exhibited perforation of the vestibular cortex, lingual perforation opposite tooth 46, and root resorption involving teeth 35, 34, and 41. The inferior alveolar canal was displaced but remained intact and identifiable.

The multilocular lesion of the symphysis was primarily suggestive of an OK. Differential diagnoses included ameloblastoma, odontogenic myxoma, radicular or residual cyst, and central giant cell granuloma. A chronic dental infection was also considered.

Following a multidisciplinary consultation involving infectious disease specialists, endodontists, orthodontists, maxillofacial surgeons, and orthopedic surgeons, an initial phase of endodontic treatment was carried out on teeth 36 to 46. This was followed by removal of the orthodontic appliance and placement of a bonded retainer from teeth 33 to 43. All therapeutic options were discussed with the patient, who subsequently provided written informed consent for the proposed management.

**Figure 2:**
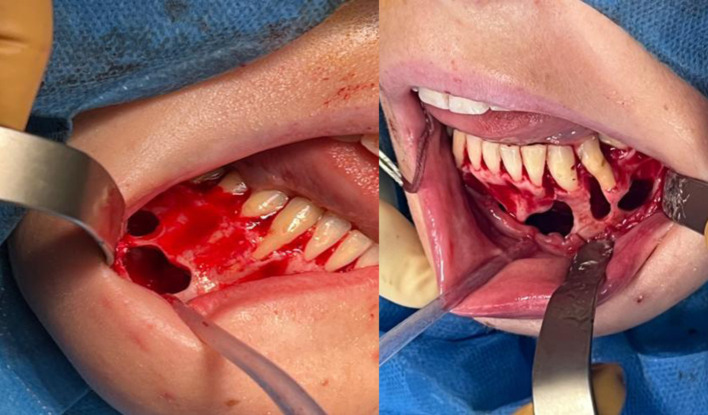
Surgical site after tumor enucleation.

The surgical procedure, performed as an outpatient intervention under general anesthesia, began with a mucoperiosteal incision extending from tooth 46 to tooth 36, enabling elevation of a wide full-thickness flap and identification of the mental and inferior alveolar nerves. The vestibular cortex, which was extremely thin and perforated in several areas, was accessed through a peripheral osteotomy, maintaining a 5 mm bony safety margin around the perforations. The tumor was gradually dissected, aided by the injection of pressurized saline between the bony wall and the cystic capsule. The lingual transcortical extensions were carefully released using blunt scissors to preserve adjacent structures and minimize the risk of lingual mucosal perforation (Fig. 2).

The excised tissue was submitted for histopathological and bacteriological analysis. Careful peripheral curettage was then performed until a macroscopically healthy bone bed was obtained, followed by thorough irrigation of the surgical site with 10% povidone-iodine and a final rinse with saline solution. Given the well-defined radiographic appearance of the lesion, suggestive of a cystic lesion compatible with an OK, and its surgical accessibility, we proceeded directly to excision in order to both obtain histopathological confirmation and achieve definitive treatment in a single-stage procedure.

**Figure 3:**
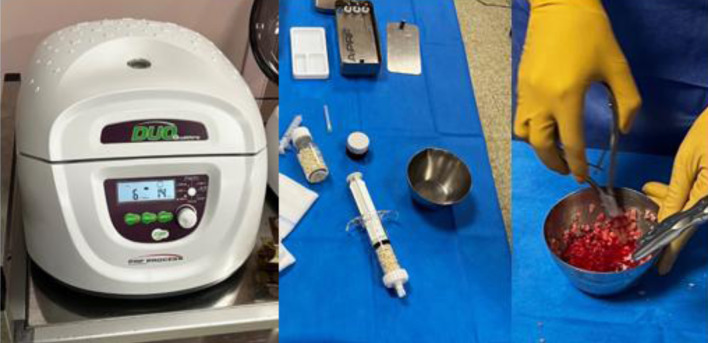
Preparation of the sticky bone.

**Figure 4:**
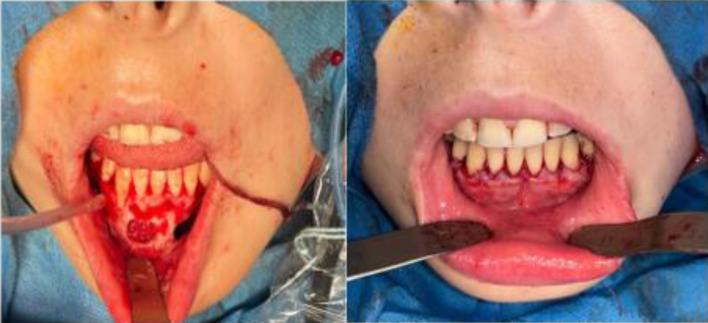
Graft in place (left) and closure of the surgical site (right).

This approach was also motivated by the desire to avoid delays in management and to reduce the risk of infection or alteration of the cystic content, which could potentially complicate histopathological interpretation in the event that lesion aspiration had been performed prior to surgery for histological confirmation. Before closure, the bone defect was reconstructed using a composite graft composed of liquid platelet-rich fibrin (PRF), obtained intraoperatively from the patient’s blood and allogeneic cancellous bone particles (24 mL of granules and 4 mL of BIOBANK^©^ bone powder of human allogeneic cancellous bone particles from a certified tissue bank), producing a cohesive fibrin-rich composite graft and commonly referred to as sticky bone [6], and subsequently covered with PRF membranes (Fig. 3). A total of 25 blood tubes (6 mL each) were collected from the patient during the procedure. A deep PRF membrane was also placed at the base of the cavity to isolate the lingual perforation and protect the inferior alveolar nerve. The graft was placed without compression and entirely covered with additional PRF membranes to ensure stable three-dimensional containment. Mucosal closure was achieved with 4-0 absorbable sutures without tension, and no additional dental stabilization devices were required (Fig. 4).

Bacteriological analysis revealed the presence of *Actinomyces odontolyticus*, indicating chronic intracystic superinfection. In accordance with the recommendations of the French Society of Infectious Pathology (SPILF – Societe de Pathologie Infectieuse de Langue Française) [7], antibiotic therapy was adjusted on postoperative day 15, increasing from 2 to 6 g/day of amoxicillin, accompanied by probiotic supplementation (Ergyphilus^®^).

The immediate postoperative course was uncomplicated. However, during the first postoperative month, the patient experienced significant fatigue, requiring a 15-day leave from work. A marked improvement in fatigue and psychological well-being was observed beginning in the third week.

Follow-up examinations on days 1, 7, and 21, and at 1 and 2 months, demonstrated satisfactory mucosal healing without suppuration or dehiscence. No sensory disturbance was noted on the right side. On the left side, postoperative partial chin dysesthesia was observed, with gradual improvement during the first month. The patient reported the resolution of anterior swelling and the reappearance of the labiomental groove as a notable aesthetic benefit, without functional impairment.

**Figure 5:**
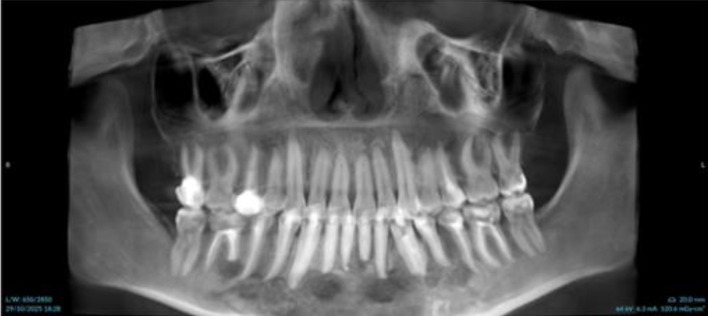
The 3-month cone beam computed tomography follow-up.

Histopathological analysis confirmed an MOK, showing parakeratinized epithelium with satellite microcysts and no cytonuclear atypia. Bacteriological findings again identified *Actinomyces odontolyticus*, consistent with chronic intracystic superinfection. The 3-month CBCT follow-up demonstrated homogeneous ossification, preservation of the mandibular nerve, and absence of recurrence (Fig. 5).

## Discussion

OKs have undergone several terminological changes across the WHO classifications. Initially considered as keratocystic odontogenic tumors due to their aggressive behavior and high recurrence rate, they were reclassified in 2005 as OKs, and then reclassified again in 2017 into the category of odontogenic cysts and lesions, confirming their cystic rather than neoplastic nature. The 2022 WHO classification maintains this terminology and further specifies their histopathological and radiological features [3]. From a pathogenetic standpoint, these lesions are associated with alterations in the PTCH1/SHH signaling pathway, explaining their growth potential and tendency for recurrence [8]. Epidemiologically, they account for approximately 10–12% of odontogenic cysts and occur mainly in young adults with a slight male predominance [4]. Finally, an association with Gorlin-Goltz syndrome (basal cell nevus syndrome) is well established, particularly in multiple or recurrent cases [9].

Management of anterior multilocular forms is especially challenging, as they are frequently associated with cortical perforations and root resorption, complicating excision and increasing the risk of recurrence. In this case, the symphyseal location rendered the radiological presentation unusual, underscoring the need for careful diagnostic evaluation and meticulous surgical planning.

Management strategies for MOK vary according to lesion aggressiveness, location, and recurrence risk. Conservative approaches include marsupialization, decompression, and enucleation, often supplemented by peripheral osteotomy to minimize recurrence while limiting surgical morbidity [10–12]. More invasive procedures, such as marginal or *en bloc* resection, are typically reserved for large, multilocular lesions that are recurrent or associated with cortical perforation or root resorption. Mandibular marginal resection enables local control of the lesion while preserving bone continuity, thereby minimizing functional and aesthetic consequences [13, 14]. The choice of surgical techniques must be individualized, taking into account the lesion’s size, its proximity to the inferior alveolar nerve, and any associated infectious context.

In our case, a conservative mandibular marginal resection was preferred, allowing complete excision of the lesion while maintaining bone continuity. This approach differs from segmental resection, which, despite its oncological efficacy, carries significant functional and aesthetic morbidity. The combination of resection with healthy margins, intraoperative identification of the inferior alveolar nerve, and immediate biological reconstruction achieved optimal anatomical and functional outcomes, with no persistent sensory deficits or compromise of mandibular architecture.

For reconstruction of the bone defect, the use of sticky bone provided three-dimensional stability of the graft and a sustained release of growth factors, including vascular endothelial growth factor (VEGF), platelet-derived growth factor (PDGF), and transforming growth factor-beta (TGF-β). This dense fibrin network promotes angiogenesis and bone maturation and contributes to the reduction of infectious complications through the immunomodulatory action of leukocytes. Several studies have demonstrated that PRF improves the quality of newly formed bone, reduces early graft resorption, and decreases postoperative morbidity by enhancing vascularization and mucosal healing [15].

Nevertheless, various bone regeneration strategies can be considered depending on the defect volume and the local biological environment. The combination of PRF with allogeneic graft material stimulates early bone formation and limits resorption between the third and fourth months, while reducing the risk of infection [16, 17]. Autologous bone, particularly from iliac crest grafting, remains the gold standard for extensive reconstructions due to its direct osteogenic potential; however, its significant donor-site morbidity restricts its routine use in oral surgery [18].

In addition, certain lesions may benefit from adjuvant therapies, such as Carnoy’s solution [19] or liquid nitrogen [20], aimed at reducing the risk of recurrence after enucleation. Another promising approach is the induced membrane technique, also known as the “Masquelet” procedure, originally described in orthopedic surgery [21]. Recent studies have demonstrated that the newly formed membrane is highly vascularized and rich in osteogenic mediators, including VEGF, bone morphogenic protein (BMP), and TGF-β, providing a bone regeneration potential comparable to that achieved with guided tissue regeneration [22, 23]. As demonstrated in this case, adapting this approach to the mandible could represent a relevant biological option for the reconstruction of large post-cystectomy or post-tumor bone defects.

Within this context, sticky bone emerges as a particularly attractive intermediate solution: it is less invasive than autologous bone grafting, more stable than simple particulate grafts, and compatible with immediate reconstruction in mandibular defects requiring reliable three-dimensional restoration.

This case also highlights the critical importance of a truly multidisciplinary approach, integrating the expertise of infectious disease specialists, endodontists, orthodontists, oral surgeons, and orthopedic surgeons. Such close collaboration ensured the safety and success of each treatment stage: preliminary infection control, endodontic therapy, functional orthodontic preparation, followed by biological sterilization and surgical resection under optimal conditions. The complementary nature of these skills was essential for achieving stable bone reconstruction and favorable anatomical and functional outcomes.

The main limitation, however, remains the relatively short follow-up period, particularly given the high risk of recurrence associated with MOK. Long-term clinical and radiological monitoring is therefore essential to confirm the enduring effectiveness of the adopted therapeutic strategy.

### Conclusion

Multidisciplinary management of a symphyseal MOK enabled complete excision with immediate reconstruction using sticky bone. The 3-month clinical and radiological outcome was favorable, with no signs of recurrence and satisfactory sensory recovery. The identification of *Actinomyces odontolyticus* necessitated prolonged antibiotic therapy, underscoring the importance of systematic bacteriological analysis in such lesions. Given the high risk of recurrence associated with MOKs, close radiological follow-up remains essential. The use of autologous biomaterials, particularly PRF in the form of sticky bone combined with PRF membranes, offers a promising approach for immediate biological reconstruction, promoting rapid tissue regeneration while reducing the risk of infection.

## Data Availability

The authors declare that data supporting the findings of this study are available within the article.
